# Drug Repositioning for Cancer Therapy Based on Large-Scale Drug-Induced Transcriptional Signatures

**DOI:** 10.1371/journal.pone.0150460

**Published:** 2016-03-08

**Authors:** Haeseung Lee, Seungmin Kang, Wankyu Kim

**Affiliations:** Ewha Research Center for Systems Biology, Division of Molecular & Life Sciences, Ewha Womans University, Seoul, Korea; National Institute of Genomic Medicine, MEXICO

## Abstract

An *in silico* chemical genomics approach is developed to predict drug repositioning (DR) candidates for three types of cancer: glioblastoma, lung cancer, and breast cancer. It is based on a recent large-scale dataset of ~20,000 drug-induced expression profiles in multiple cancer cell lines, which provides i) a global impact of transcriptional perturbation of both known targets and unknown off-targets, and ii) rich information on drug’s mode-of-action. First, the drug-induced expression profile is shown more effective than other information, such as the drug structure or known target, using multiple HTS datasets as unbiased benchmarks. Particularly, the utility of our method was robustly demonstrated in identifying novel DR candidates. Second, we predicted 14 high-scoring DR candidates solely based on expression signatures. Eight of the fourteen drugs showed significant anti-proliferative activity against glioblastoma; *i*.*e*., ivermectin, trifluridine, astemizole, amlodipine, maprotiline, apomorphine, mometasone, and nortriptyline. Our DR score strongly correlated with that of cell-based experimental results; the top seven DR candidates were positive, corresponding to an approximately 20-fold enrichment compared with conventional HTS. Despite diverse original indications and known targets, the perturbed pathways of active DR candidates show five distinct patterns that form tight clusters together with one or more known cancer drugs, suggesting common transcriptome-level mechanisms of anti-proliferative activity.

## Introduction

Drug repositioning (DR) refers to the identification of novel indications for existing drugs [1] and is considered an effective route for drug development because it reduces costs and bypasses safety concerns. However, discovering novel indications with DR is highly challenging, even with well-established high-throughput screening (HTS), because of the numerous combinations of both assays and drugs [2]. Due to these limitations, most repositioned drugs have been serendipitously developed. *In silico* DR is considered an alternative and efficient route to establish novel connections between diseases and existing drugs [3,4]. Advances in systems pharmacology approaches and the growth of drug-target information have increased the success of *in silico* DR [5,6]. A broad range of datasets has been utilized, such as sets related to chemical structure [7,8], drug-target relationship [9], and phenotypic information including drug side effects [10–14]. For example, Cheng *et al*. identified simvastatin and ketoconazole as potent anti-proliferative agents in breast cancer by analysing a drug-target network [15]. Particularly, large-scale chemical genomics data, such as the Connectivity Map or CMAP [16], have provided rich information on the modes-of-action of drugs that are reflected in the transcriptomic responses due to chemical perturbation. However, the relative utilities of different datasets were not rigorously evaluated because the compound set of CMAP was not large enough for integrative statistical analysis.

The latest version of CMAP consists of gene expression profiles of five cell lines treated with ~1,300 compounds, from which many *in silico* DR methods were developed using this dataset either alone or in combination with other information [17–25]. The negative correlation of gene expression with a disease led to the identification of topiramate for the treatment of inflammatory bowel disease (IBD) and cimetidine for the treatment of lung adenocarcinoma [19,20]. Iskar *et al*. further revealed the modes of action for multiple drugs using drug-induced expression modules conserved between humans and rats. Recently, a similar but highly expanded version of a chemical genomics dataset was publicly released by the NIH LINCS program (Library of Integrated Network-based Cellular Signatures). This dataset consists of gene expression signatures and protein binding, cellular phenotypic, and phosphoproteomic profiles due to chemical or genetic perturbation. Specifically, it produced the gene expression profiles of ~1,000 landmark genes (L1000) in response to >20,000 chemical perturbations across many cell lines. Additionally, they inferred transcriptome-level expression profiles of ~20,000 genes computationally using the 1,000 landmark genes [26].

In this study, we adopted an integrative approach for *in silico* DR using the expression signature (E) derived from the recent large-scale, chemical genomics dataset (LINCS) as well as chemical structure (S) and target signatures (T). Next, we applied our method to infer DR candidate anti-cancer drugs for glioblastoma, lung cancer, and breast cancer. We focused on the ability to identify novel DR candidates that are not structurally related to known anti-cancer drugs because structural analogues may be inferred easily by other structure-based methods [27,28]. The LINCS dataset covers a sufficiently large number of compounds that allowed the unbiased evaluation of the predictive power of each signature. We then predicted novel DR candidates for glioblastoma. The high-scoring candidate drugs were experimentally validated using cancer cell lines and patient-derived primary cells. The LINCS dataset also enabled us to interpret the mode of action of the validated DR candidates.

## Materials and Methods

### Known drug set and compound-target information

The known drug set (KD set or *gold standard*) was extracted from several public databases of DrugBank [29], CTD [30], PubChem [7], and KEGG DRUG [31] for glioblastoma, lung cancer, and breast cancer. Additionally, we manually curated active compounds and drugs from PubMed by searching abstracts that explicitly described anti-cancer activities for these three types of cancer (S1 Table). A typical query included several terms, such as ‘apoptosis’, ‘proliferation’, ‘cell growth’, ‘cytotoxicity’, ‘anti-cancer’, ‘cytotoxicity’, and ‘increased survival’ after treatment. The compound-target information was collected from 10 public data sources: DrugBank [32], KEGG [31], MATADOR [33], TTD [34], KiDB [35], BindingDB [36], ChEMBL [37], WOMBAT [38], CTD [30], and DCDB [39]. We included only the compound-target interactions supported by directed evidence, such as compound-target binding, activation, inhibition, and reaction at a reasonably high affinity (*e*.*g*., Ki < 10 nM). Throughout the analysis, compounds and protein targets were mapped to NCBI PubChem Compound Identifiers (CID) and Entrez Gene ID as standard identifiers, respectively.

### Expression signatures for the target diseases

The expression profiles for the target diseases were downloaded from either the TCGA data portal (https://tcga-data.nci.nih.gov) or NCBI GEO [40] that included both normal and disease conditions. The glioblastoma datasets of 200 patients was obtained from TCGA that were divided into four canonical subtypes—classical, mesenchymal, proneural, and neural. Four distinct expression signatures were extracted for the corresponding subtypes. For lung and breast cancer, 11 and 16 microarray datasets were obtained, respectively. The 11 lung cancer datasets consisted of GDS1761, GDS1312, GDS2771, GSE5364, GSE7670, GSE10072, GSE10799, GSE1987, GSE2088, GSE1037, and GSE11969. The 16 breast cancer data sets consisted of GDS817, GDS820, GDS823, GDS1761, GDS1925, GDS2250, GDS2617, GDS2618, GDS2635, GDS2739, GSE5364, GSE10780, GSE15852, GSE16443, GSE17072, and GSE20266. All microarray datasets were processed and normalized using the SAM package [41]. The DEGs were extracted as expression signatures using FDR<0.05 as a cutoff.

### Expression signatures from the LINCS dataset

The LINCS dataset included an extensive catalog of gene-expression profiles generated by the Library of Integrated Network-based Cellular Signatures (LINCS) project from 59 human cancer cells in response to ~20,000 chemical perturbations. The LINCS team has produced the expression profiles of 1,000 landmark genes using a high-throughput Luminex-based assay [42]. The whole transcriptomic profiles for ~20,000 genes were deduced from the measured expression values of the 1,000 landmark genes. The expression signatures for the compounds were downloaded from the LINCS project page (www.lincsproject.org) that consisted of the 100 most up- and down-regulated genes in response to each compound.

### Cell Viability Assay

The compounds used for the cell viability assay were purchased from Sigma-Aldrich and Enzo Life Science. Both the glioblastoma cell lines (A172, T98G, U251, and U87) and primary cells (GBL cells) were cultured in DMEM high-glucose cell culture medium (HyClone; SH30243.01) supplemented with 10% fetal bovine serum, 1% penicillin/streptomycin solution, and 30 μg/ml Plasmocure (InvivoGen, ant-pc) reagent to prevent mycoplasma contamination.

To measure the cytotoxic activity of the candidate drugs, the metabolic activity of the viable cells was measured using WST reagent (EZ-Cytox, DoGEN) in 96-well plates. The number of seeded cells was adjusted according to the growth rate of each cell type (100–500 cells/well). Twenty-four hours after seeding, the cells were treated with drugs at a concentration of 10 μM and further cultured for 72 h at 37°C. One-tenth of the medium volume of WST reagent was added to the cells, and the absorbance was measured at 450 nm after 2 h using a SpectraMax 190 microplate reader (Molecular Devices). The experiments were repeated five times.

### Pathway enrichment analysis

KEGG pathways were downloaded from the MSigDB database (http://www.broadinstitute.org/gsea/msigdb), which consist of 186 gene sets representing various biological processes. Since many disease-related pathways are redundant with other signalling pathways (e.g. *KO05200*:*Pathways in cancer* include *KO04310*:*Wnt*, *KO04210*:*Apoptosis*, *KO04115*:*p53 signaling*, etc.), we excluded such 28 pathways and the remaining 158 pathways were used. The significance of enrichment was calculated using the hypergeometric test. The LINCS dataset contains multiple signatures for the same drug from different cells and conditions, where the harmonic mean of the corresponding p-values was taken as its representative p-value. The adjusted q-value was then calculated using the Benjamini-Hochberg method. The distances between drugs were calculated as the cosine distance of–log p-value.

## Results

### Data collection and processing

First, we compiled a list of known drugs (KD set) as the benchmark for the three types of cancer (glioblastoma, lung cancer, and breast cancer) from four public databases as well as by manually curating 243 publications (S1 Table). We considered only the compounds that were explicitly stated as being active against the target disease—*e*.*g*., drug X induces apoptosis, inhibits proliferation, or shows cytotoxic activity. The KD set consisted of 132, 216, and 256 compounds for the treatment of glioblastoma, lung cancer, and breast cancer, respectively (Figure A in S1 File). Additionally, we collected an extensive list of 1,155 compounds (cancer drug set or CD set) that were reported to show the anti-proliferative activity against any other type of cancer including all the drugs in the KD set.

Next, the structural signature was extracted as 1,024 bits of FP2 chemical fingerprints implemented in Open Babel [43] for compounds (S_CPD_) or known drugs (S_KD_). The target information was collected from 10 public databases comprising 342,311 compound-target interactions among 205,570 unique compounds (Figure A in S1 File). The sets of genes associated with each compound or drug were then generated and served as the target signatures (T_CPD_, T_KD_). The expression signature (E_CPD_, E_KD_) was downloaded from LINCS and consisted of the differentially expressed genes (DEGs) by chemical perturbation[26]. Multiple signatures for a single compound were common because expression signatures were generated in various cell lines of different origins. Additionally, the LINCS dataset included a significant fraction of redundant compound IDs and some generic compound names, 2D structures, and/or stereoisomers of the same molecular formulae were not distinguishable between different data sources. Therefore, the signatures of these similar compounds were merged together after converting them to the canonical SMILES format, although stereoisomers may show different pharmacological activities and some were originally assigned different compound IDs by LINCS. We note that ID mapping of a stereoisomer was very rare between two distinct stereoisomers, but was done mostly with generic name or SMILES without 3D information. The resulting number of unique compounds was reduced to 8,860. We defined a *core set* of 2,250 compounds for which all three types of signatures (S, T, and E) were available. The intersection of the core set and CD set was 304 drugs (Figure A in S1 File). Similarly, we also generated disease expression signatures (E_DIS_) for glioblastoma (4 sets), lung cancer (11 sets), and breast cancer (16 sets) from TCGA [44] or public microarray datasets from GEO. The detailed procedure is described in the Materials and Methods section.

### Overview of the analysis

We developed a series of classifiers to predict DR candidate drugs for the treatment of glioblastoma, lung cancer, and breast cancer. Our method utilizes three types of signatures that are derived from chemical structure (S), drug-target relation (T), and gene expression data (E). DR candidates were predicted based on the similarity of these signatures between the compounds and disease (or its known drugs). The prediction performance was thoroughly inspected in an unbiased manner using i) a conventional cross-validation scheme that utilizes known drugs (KD set) as a benchmark, ii) the 29 anti-cancer HTS datasets for 11,000–41,000 compounds, and iii) assays based on glioblastoma cancer cell lines and patient-derived primary cells.

**Fig 1:**
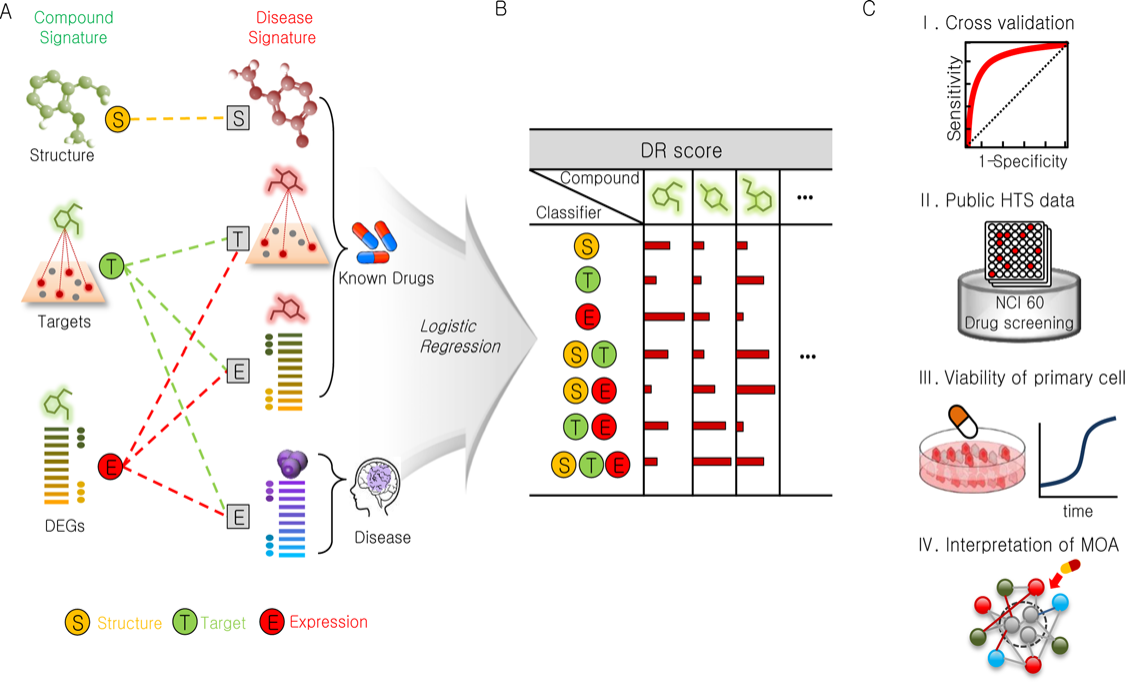
Overview of the *in silico* DR procedure. (A) The structural (S), target (T), and expression (E) signatures for each compound (circles on the left) and disease (squares on the right) were compared. The associations are indicated by dashed lines in three categories (S: yellow, T: green, E: red) depending on the type of compound signature. (B) In total, seven different classifiers were constructed based on the similarity between the compound and the target signature or their combinations (S, T, E, ST, SE, TE, and STE). The DR scores were calculated using a series of classifiers based on a logistic regression with the known drug set (KD set) used as a benchmark. (C) The performance was evaluated using three independent datasets: I) the mean AUC of 100 rounds of 3-fold cross validation, II) comparison with the 29 sets of NCI-60 DTP human tumor cell line HTS data, and III) experimental validation of anti-proliferative activities using cancer cell lines and primary cells. A pathway-level interpretation of the drug mode of action was performed for active DR candidates for glioblastoma (IV).

The work described herein consisted of three stages: 1) building association signatures, 2) constructing a series of classifiers, and 3) evaluating the prediction performance. The aim of the first stage was to associate compounds and a target disease (or its known anti-cancer drugs) based on the similarity of the three signature types (Fig 1A). In total, seven distinct types of associations that were independent of each other were established. First, a compound was predicted as a DR candidate based on its structural similarity to the known drugs (S_CPD_-S_KD_). The expression (E) and target (T) signatures essentially are a list of genes that could be associated by any method for gene set enrichment analysis. Therefore, we could generate six additional types of associations between the compounds (T_CPD_, E_CPD_) and disease (T_KD_, E_KD_, E_DIS_). We used the *Tanimoto coefficient* as a measure of signature similarity because all signatures can be represented as a binary vector of 0s and 1s—*i*.*e*., the presence or absence of genes or structural fingerprints. Because multiple signatures are allowed for a single compound and disease, we calculated the mean of the Tanimoto coefficient values for a given compound-disease pair.

The second stage involved the construction of a series of classifiers to predict DR candidates (Fig 1B). To compare the performance of each signature, seven classifiers were constructed using a logistic regression. The resulting classifiers used a single (S, T, E), combination of the two (ST, SE, TE), or all three types (STE) of compound signatures. Finally, the prediction performance was thoroughly inspected in an unbiased manner using i) a conventional cross-validation scheme that utilizes known drugs as the *gold standard*, ii) the 29 large-scale HTS datasets for anti-proliferative activity, and iii) assays that use both established glioblastoma cell lines and patient-derived glioblastoma primary cells. We further interpreted the potential modes of action of drugs using pathway enrichment analysis followed by comparisons with other cancer drugs.

### Performance evaluation using the known drug (KD) dataset

To ensure unbiased evaluation, we limited our analysis to the core set of 2,250 compounds so that different classifiers can be compared using exactly the same benchmark. The core set included 79, 100, and 132 known anti-cancer drugs (KD set) as benchmarks for glioblastoma, lung cancer, and breast cancer, respectively. The negative set (1,946 compounds) was prepared by excluding the CD set from the core set.

**Fig 2:**
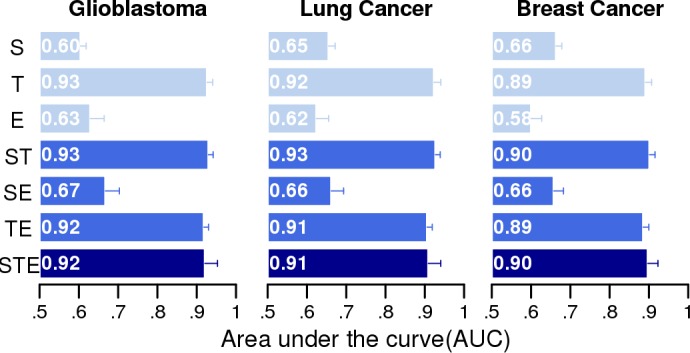
Evaluation of prediction performance using the known drug (KD) set as a benchmark. The classifiers using a single type of signature (S, T, and E) and their combinations (ST, SE, TE, and STE) were evaluated based on the AUCs of the ROC curve for glioblastoma, lung cancer, and breast cancer. The AUC values were calculated by averaging 100 rounds of 3-fold cross validation.

As stated in the previous section, we constructed seven different classifiers based on a single signature (S-, T-, and E-classifier) or combination of multiple signatures (ST-, SE-, TE-, and STE-classifier). Their relative performances were evaluated using 100 rounds of three-fold cross-validation schemes. The classifiers were based on a logistic regression that automatically weighs the component features to yield a unified prediction score—the *DR score*—scaled from zero to one. The target-based classifiers (T) perform better than the other classifiers (S, E) in identifying known drugs (KD core set) in all three types of cancer (Fig 2). This trend was consistently observed in combination with other signatures (ST, TE > SE). We reasoned that the targets of known drugs (KD) tend to be better characterized than other compounds, and target-based classifiers may consequently favor known drugs. Therefore, we further evaluated the classifiers in a more unbiased manner in the following sections.

### Evaluation in comparison with public anti-cancer HTS

To avoid potential bias, we collected dozens of large-scale anti-cancer HTS assays from PubChem [45]. They consisted of eight, thirteen, and eight HTS assays for GBM, lung cancer, and breast cancer cell lines, respectively (Table A in S1 File). These assays all used different cell lines and were reasonably distinct from each other in terms of both assayed and hit compounds—*e*.*g*., only a 20% hit overlap was observed between the two assays for GBM (AID57, AID59) (Figure B in S1 File). Again, we limited our evaluation to the assayed compounds that also belonged to the core set. To focus on the ability to predict novel DR candidates, known drugs (KD) were excluded from the hit compounds. The remaining hits of each assay were divided into two categories: i) anti-cancer hits that belonged to the CD set or had two or more structural analogs in the CD set with a Tanimoto coefficient > 0.7, and ii) novel hits that were not included in the former set. Therefore, the anti-cancer hits represented those that could be readily predicted based on structural similarity and the novel hits, which were the difficult cases and are the focus of this study.

**Fig 3:**
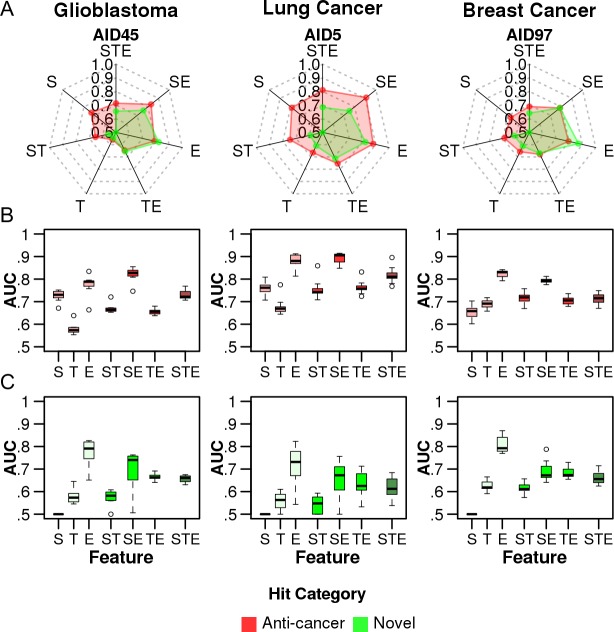
Performance evaluations using the public anti-cancer HTS dataset as a benchmark. The seven classifiers (S, T, E, ST, SE, TE, and STE) were evaluated based on the AUCs of the ROC curve for glioblastoma, lung cancer, and breast cancer. Only compounds in the core set were evaluated. The AUC values were calculated by averaging 100 rounds of 3-fold cross validation. (A) Typical examples of performance evaluation using the HTS data set for glioblastoma (AID45), lung cancer (AID5), and breast cancer (AID97). The AUCs were independently calculated using two distinct sets of hit compounds as a benchmark (or positives)—i) the hit compounds of known anti-cancer activity (red lines) and ii) the novel hits (green lines). The distribution of AUCs using (B) the compounds of known anti-cancer activity as a benchmark, and (C) the novel hits as a benchmark.

Interestingly, the performance of each signature was in contrast to the result of the previous evaluation based on the KD set. The expression-based classifier (E) best predicted anti-cancer hits in all three cancers (Fig 3A). By contrast, the target-based classifier (T) performed worst. This trend was more obvious in the prediction of novel hits (Fig 3B). The classifiers based on structure (S) or target signature (T) were essentially unable to predict novel hits (median AUC = 0.41~0.62), whereas the expression signature (E) performed reasonably well (median AUC = 0.73~0.79, Fig 3B). This trend was consistently observed in combination with other signatures (SE > TE > ST). Notably, the single signature (E) consistently performed better alone than in combination with other signatures (SE, TE) for all three cancer types. Overall, the expression signature (E) was more informative than the structure (S) or target (T) signatures among the HTS datasets for the three different cancers. The data also suggested that previous evaluations of any *in silico* DR could be biased if known drugs were used as the only benchmark set, particularly if the method was highly dependent on target information. Notably, focusing on the core set retains only a small set of compounds for evaluation, but this approach remained sufficient for unbiased statistical tests using the same set of compounds (Table A in S1 File). The evaluation results for all the compounds in the HTS dataset showed essentially the same trend (Figure C in S1 File).

### Experimental validation of DR candidate drugs for glioblastoma

Glioblastoma multiforme (GBM) is the most common and aggressive form of brain cancer showing a highly poor prognosis despite concurrent or sequential chemo-radiotherapy [46]. Temozolomide (TMZ) is a first-line chemotherapeutic agent for the treatment of GBM. As an alkylating agent, TMZ transfers methyl groups to the purine bases of DNA, causing single- and double-strand DNA breaks and subsequent apoptotic cell death. Most studies reported that chemotherapy conferred a limited overall survival benefit to GBM patients. The median time to recurrence was only 6.9 months after standard treatment [47]. Therefore, the need for more effective drugs is clear and unmet.

**Fig 4:**
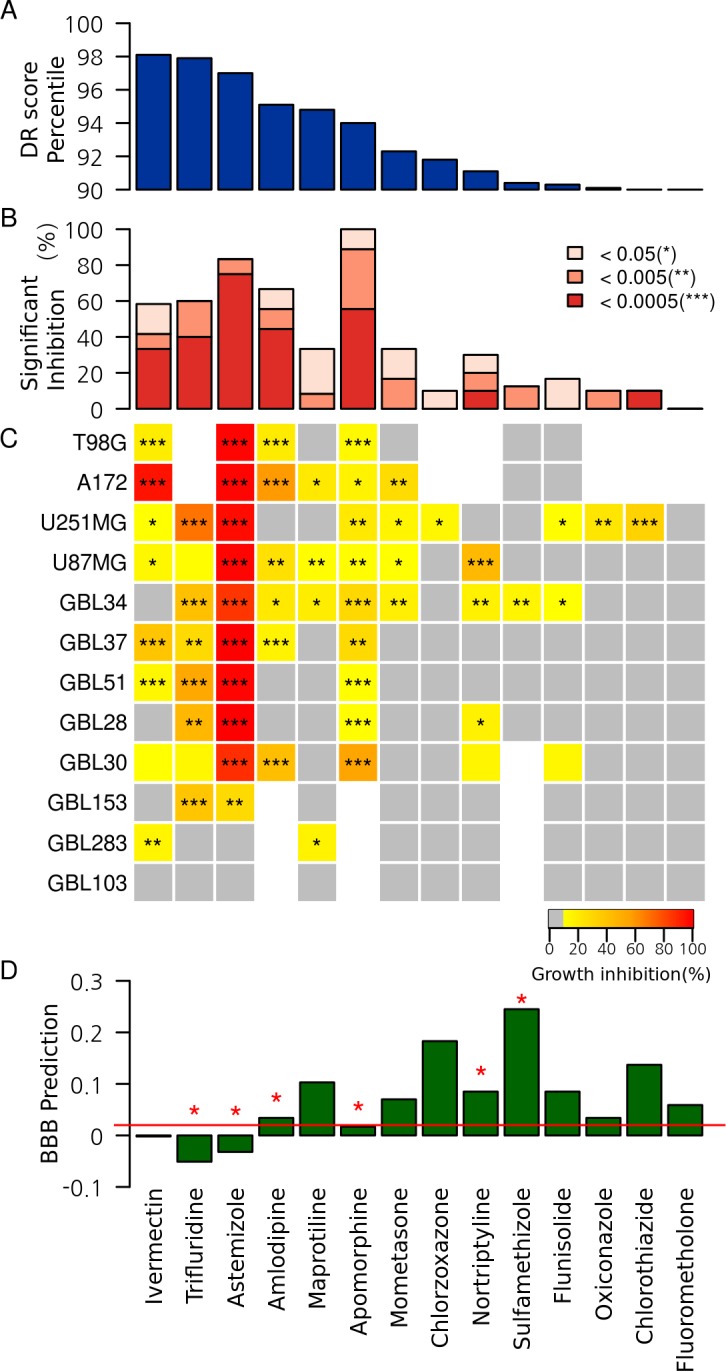
The high-scoring DR candidates for glioblastoma among the FDA-approved drugs that were predicted based only on the expression signatures. (A) DR scores, (B) the fraction of significantly inhibited cells summarizing the results of (C), (C) the anti-proliferative activities (% growth inhibition) for the four glioblastoma cell lines (four cell lines of TG98, A172, U251MG, and U87MG) and the eight patient-derived primary cells (the GBLs) at 10 μM, (D) in silico prediction scores for BBB transport based on http://www.cbligand.org/BBB. The red asterisk indicates experimental support for passing the BBB according to the literature. Overall, anti-proliferative activities across glioblastoma cells strongly correlated with the rankings by the DR score. Most DR candidates were shown to be able to pass the BBB.

Here, we listed DR candidates for GBM and experimentally tested their anti-tumor activities solely based on the expression signature. First, the 8,860 compounds were ordered by the DR score, *i*.*e*. the percentile rank of the predictions by the same classifier (E) as described in the previous section. Next, we applied several filters: a) FDA-approved; b) DR score >0.9; and c) indication to pass brain-blood barrier (BBB) either by literature or by *in silico* prediction at http://www.cbligand.org/BBB. One high-scoring candidate (ivermectin, DR score = 0.98) was also included despite being indicated not to pass BBB. The final 14 DR candidates were selected, and their anti-tumor activities were tested using four GBM cell lines and eight patient-derived primary cells. Our DR scores correlated well with the anti-tumor activities among the cell lines tested in terms of both anti-proliferative activity and the fraction of significant growth inhibition (Fig 4A and 4B). Eight of the 14 candidates were significant hits in three or more GBM cells at 10 μM. Notably, the top seven candidates showed strong anti-tumor activity, corresponding to an approximately 20 fold hit enrichment from ~5% hit rate of conventional high-throughput screening.

We surveyed the literature for reports concerning the anti-cancer activity of the eight active DR candidates, as summarized in Table 1. We found that four drugs (ivermectin, astemizole, nortriptyline, and apomorphine) were previously reported to show anti-tumor activity in brain cancer. Three drugs (trifluridine, amlodipine, and maprotiline) demonstrated anti-tumor activity in other cancers but not in glioblastoma. Mometasone is a corticosteroid and is considered a novel drug for cancer because it has not been reported to show anti-tumor activity in any type of cancer. Corticosteroids have been used to reduce peritumoral edema and chemotherapy-associated side effects, such as pain, nausea, and vomiting [48]. All active DR candidates except ivermectin have been shown to be able to pass the BBB in either literature reports or via *in silico* prediction.

**Table 1:** The list of active DR candidates.

Name	DR Score	Original Indication	Targets	BBB Permeability	Cancer Indication
Ivermectin	0.98	antiparasitic	GABRB3, GLRA3, CYP3A4, ABCB1, ABCC1, ABCC2, ABCG2	-	**Glioblastoma,**Lung,Colon,melanoma [49],ovarian [50]
Trifluridine	0.98	antiviral	PARP1, CASP3, CASP8, CASP9, CTSB, TYMS	O [51]	colorectal [52]
Astemizole	0.97	antihistamine	CYP2D6, CYP2J2, CYP3A4, HRH1, ICAM1, IGF1, IL1B, KCNH1, KCNH2, KCNQ2, KCNQ3, MAPT, ABCB1, VCAM1, ABCB11	O [53]	**medulloblastoma** [54],melanoma [55]
Amlodipine	0.95	blood pressure, prevent chest pain	CACNA1C, CACNA1D, CACNA1F, CACNA1S	O [56]	epidermoid [57],breast [58]
Maprotiline	0.95	antidepressant	ADRA1A, CHRM1,CHRM2, CHRM3, CHRM4, CHRM5, DRD1, DRD2, DRD3, DRD5, HRH1, KCNH2, SLC6A2	+	Burkitt lymphoma [59],prostate [60]
Apomorphine	0.94	heroin addiction	ADRA2A, ADRA2B, ADRA2C, AVP, COMT, DRD1, DRD2, DRD3, DRD4, DRD5, GH1, HTR1A, HTR1B, HTR1D, HTR2A, HTR2B, HTR2C, JUN, MAPT, TH, CALY	O [61]	**Glioma,**Melanoma, meningioma [62]
Mometasone	0.92	inflammation	CSF2, CYP2C8, NR3C1, IL1B, IL10, PGR, ABCB1, TNF, VCAM1, ABCG2	+	None
Nortriptyline	0.91	antidepressant	SLC6A2, SLC6A4	O [63]	**glioma** [64],melanoma [65]

### Interpretation of drug modes of action for the active DR candidates

**Fig 5:**
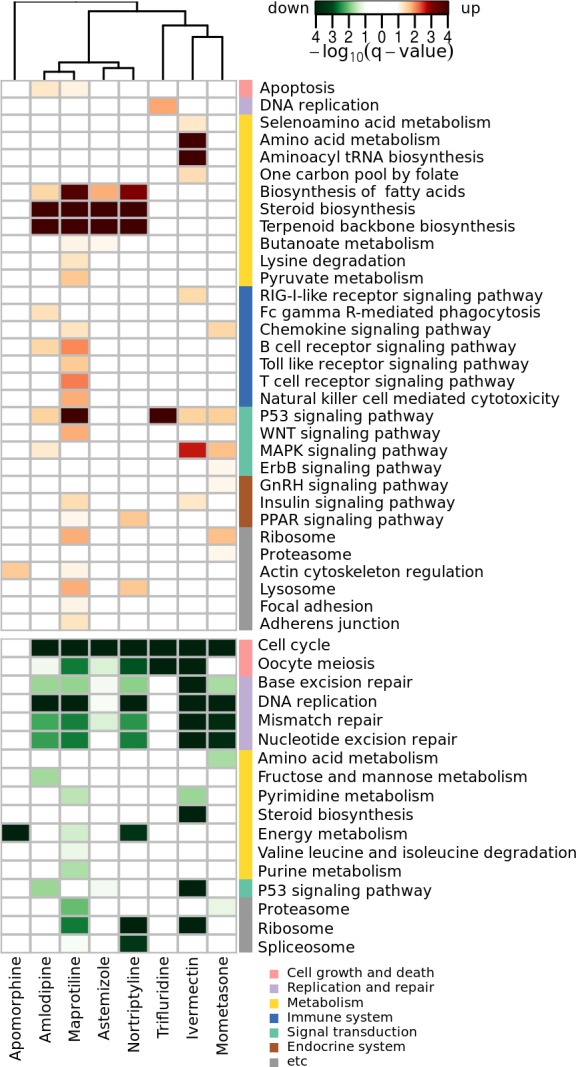
Pathway enrichment pattern of the eight active DR candidates for glioblastoma. The p-values and their adjusted q-values were calculated by hypergeometric test and the Benjamini-Hochberg method, respectively.

Drug-induced transcriptomic profiles reflect direct and indirect changes of cellular physiology in omics scale and provide rich information on the pharmacological mechanism of drugs. In order to interpret their modes of action, we performed pathway enrichment analysis of up- and down-regulated genes using KEGG pathways. Collectively, 32 and 17 pathways were significantly enriched in the up- and down-regulated genes, respectively, at a cut-off q-value<0.1 (Fig 5). Overall, the data suggest plausible mechanisms for anti-tumor activities that are shared among the DR candidates. Up-regulated pathways included apoptosis, amino acid and lipid metabolism, and tumor-suppressive P53/MAPK/WNT signaling, which suggested that the activities of anti-proliferative processes were increased. Conversely, cell proliferative pathways, such as the cell cycle, DNA replication, DNA repair, and ribosome assembly, were down-regulated.

Four drugs (amlodipine, astemizole, maprotiline, and nortriptyline) strongly increased lipid metabolism (steroid, terpenoid, and fatty acids). Accordingly, amlodipine, a calcium channel blocker, was previously reported to induce steroidogenesis [66]. Amlodipine and maprotiline moderately up-regulated apoptosis pathways that may be associated with PKD1 overexpression and caspase-3 activation, respectively [67,68]. Notably, apomorphine seemed to act via a distinct mechanism: genes associated with mitochondrial energy metabolism were strongly down-regulated without noticeable changes in apoptosis, metabolism, or DNA repair. These mitochondrial metabolic genes included genes that encode ATP synthases (ATP5O, ATP5D), cytochrome oxidases (COX8A, COX7), and NADH dehydrogenases (NADUFS8, NADUFB2). Mitochondrial dysfunction has been well established to modulate apoptosis and tumorigenesis [69]. The other two DR candidates (nortriptyline and maprotiline) also decreased mitochondrial energy metabolism. Down regulation of cell cycle genes was commonly observed among all eight DR candidates. Five drugs significantly decreased the expression of DNA repair genes. Overall, these active DR candidates all significantly perturbed multiple tumorigenic or tumor suppressive pathways, which may direct cancer cells toward anti-proliferative outcome.

**Fig 6:**
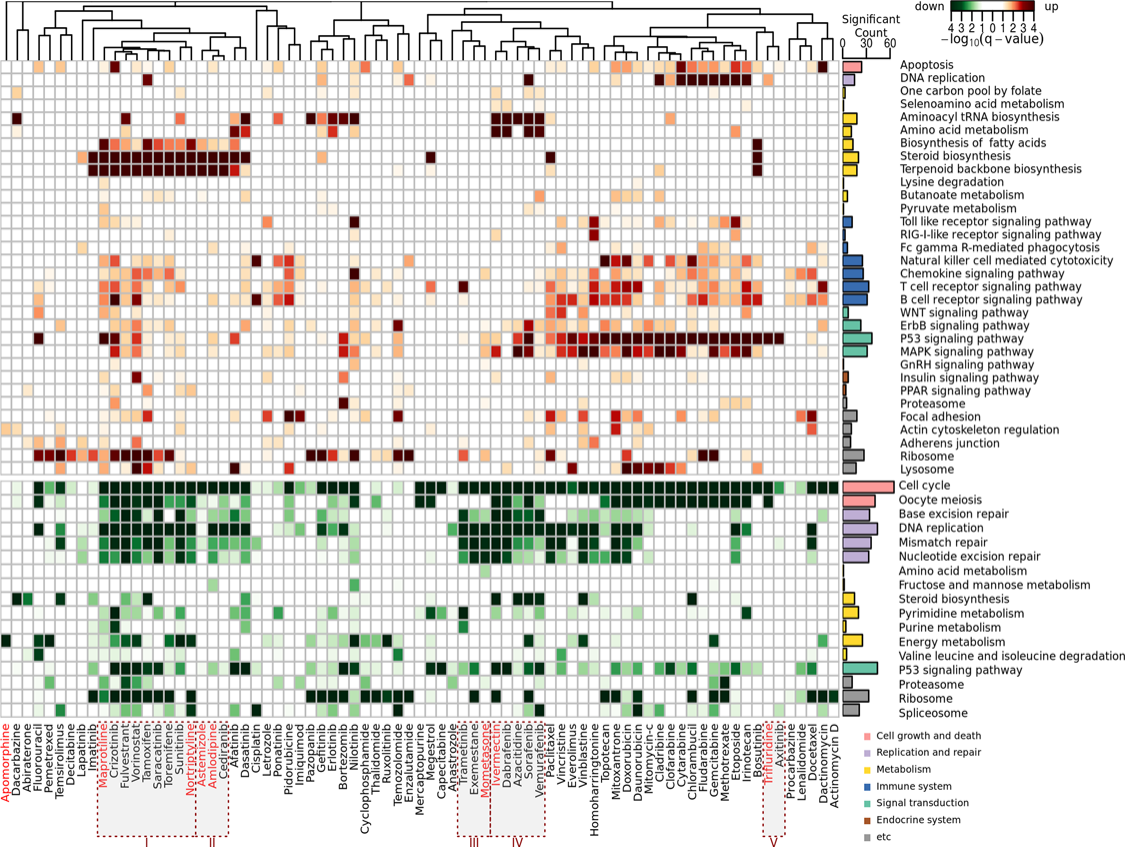
Cluster analysis of DR candidates with other cancer drugs using their pathway enrichment patterns. The eight DR candidates and 69 cancer drugs in the LINCS dataset were clustered using the 32 up- and the 17 down-regulated pathway enrichment patterns. The eight DR candidates (red) belong to five clusters (I ~ V) with 15 cancer drugs. The bar plot on the right side shows the number of significantly enriched cancer drugs (q-value<0.05) for the corresponding pathway. The significance of up- and down-regulation is presented in red and green, respectively.

We also performed a cluster analysis of the eight DR candidates together with the 69 cancer drugs included in the LINCS dataset using the enrichment pattern of the same 32 up- and 17 down-regulated pathways (Fig 6). These cancer drugs frequently up-regulate P53, MAPK, apoptosis and immune signaling. Many cancer drugs as well as our DR candidates down-regulate cell cycle-, DNA repair-, and p53 signaling pathways. Seven of the eight DR candidates show a highly similar enrichment pattern with other cancer drugs (cluster I–V in Fig 6). Four DR candidates (amlodipine, astemizole, nortriptyline, and maprotiline) and eight cancer drugs belong to two related clusters (I and II). Ivermectin is grouped with four other cancer drugs (cluster IV). Mometasone shows a pattern similar to those of trametinib and exemestane (cluster III), and trifluridine shows a pattern similar to that of axitinib (cluster V). At the pathway level, the drugs in the cluster show a similar pattern of transcriptional perturbation, although their known targets or modes of action are heterogeneous. For example, ivermectin is an antiparasitic drug that selectively binds to glutamate-gated chloride ion channels. All other cancer drugs in the same cluster have different known or canonical targets (BRAF/CRAF-dabrafenib, sorafenib, vemurafenib, DNA methyltransferase- azacitidine). Similarly, the other clusters showed a high degree of heterogeneity of known targets, suggesting that the downstream effects between the DR candidates and anti-cancer drugs are shared via the same off-target or shared regulatory events. The lists of drugs in each cluster are summarized in Table B in S1 File.

## Discussion

Although most drug development projects have been aimed to design a specific modulator for a target, polypharmacology—multiple targets for a single drug—may be more prevalent than expected. According to a recent report by Ciceri and colleagues, an unbiased screening of 628 kinase inhibitors identified 20 hits (3.2% hit rate) that also strongly inhibited BRD4 (>90% inhibition at 50 μM) [70]. Two of these kinase-bromodomain dual inhibitors (0.32% hit rate) showed an IC50 in the nano molar range. Because both kinases and BRD4 are promising targets for cancer therapy, dual inhibitors may be an effective strategy to overcome cancer heterogeneity or resistance. Extrapolating this result to thousands of druggable targets in the entire human proteome, most known and investigational drugs may have one or more off-targets. We surveyed the number of targets per compound in our drug-target dataset collected from 10 public sources. Among the FDA approved drugs, more than 60% has one or more targets. Therefore, the idea of a ‘single drug–multiple targets’ may become a general assumption in drug development, similar to the notion ‘one gene–one polypeptide’ being replaced by ‘one gene–multiple polypeptides’. Instead of striving to avoid off-targets, active exploitation of polypharmacology may improve treatments and benefit patients. Advances in chemical genomic approaches allow comprehensive understanding of drug modes of action at the whole transcriptome or proteome level, reflecting combined effects of multiple targets of a drug.

The efficient and systematic identification of clinically relevant off-targets and novel indications is a challenge. Although many *in silico* DR methods were developed, their prediction performances are not thoroughly assessed in most cases. Until recently, an *in silico* DR method was usually evaluated based on the AUC (area under the curve) of its receiver operating characteristic (ROC) curve using a collection of known or *gold standard* drugs. Experimental validation was performed in only a small number of the top-scoring DR candidates. Caution is required because the gold standard set is almost always incomplete—*i*.*e*., it includes a significant fraction of unknown false negatives or *hidden hits*. In fact, the very purpose of any *in silico* DR is to identify such *hidden hits* rather than to rediscover known drugs. Often, the ability to identify such novel DR candidates has not been thoroughly investigated or estimated using indirect information (*e*.*g*., overlap with clinical trial drugs). As we demonstrated, the benchmark dataset *e*.*g*., known drugs vs large-scale HTS dataset, can dramatically influence the performance evaluation

The CMAP dataset is the first large-scale transcriptional profile and includes >1,300 compounds. Although it triggered the development of many *in silico* DR methods that employ chemical genomic approaches, the success of these methods was mostly anecdotal due to the limited number of transcriptome-profiled compounds. The recent LINCS dataset includes the transcriptional profiles of ~10,000 unique compounds from multiple cell lines. It not only increased the compound coverage by eight fold compared with CMAP, but allowed quantitative and unbiased evaluations via cross-comparisons with multiple benchmarks, such as known cancer drugs, public HTS dataset, and wet experiments. We also managed to compare the performance of the three most frequently used data types (structure, target, and expression) for *in silico* DR, an analysis that was previously unfeasible due to the small size of the CMAP dataset. Although our analysis is limited to a simple logistic regression classifier, it strongly suggests that the expression signature was the most predictive, particularly in identifying novel DR candidates. Next, we identified eight DR candidates for glioblastoma based solely on expression signatures. Our DR score positively correlated with the anti-proliferative activities in cancer cell lines and primary cells, showing an approximately 20-fold enrichment of active hits. Notably, none of the expression signatures from LINCS were generated using glioblastoma cell lines, which demonstrated that our *in silico* DR method remains valid across different cancer types and potentially for other diseases. We reasoned that the consensus of multiple signatures from diverse cell lines provided sufficient information to overcome the heterogeneity and noise from individual signatures. Finally, we exploited large-scale, drug-induced transcriptional profiling to interpret the modes of action of our DR candidates. These candidates showed unique patterns of pathway perturbation that were shared with multiple other cancer drugs. Common patterns were observed not only among the drugs of the same target but also among drugs of different classes, which strongly indicated many unknown off-targets or cross-talk between different targets. In the post-chemical genomic era, we may expand the scope of drug repositioning from merely seeking polypharmacology (i.e. one drug for multiple targets) to broadly considering omics-scale effects by both known targets and unknown off-targets, such as the entire transcriptome, proteome, and metabolome.

## Supporting Information

S1 File Supporting figures and tables. (DOCX)

S1 Table List of known drugs collected from public databases and literature. (XLSX)
