# Supplementary material for: Drug Repositioning for Cancer Therapy Based on Large-Scale Drug-Induced Transcriptional Signatures
Source: PLoS One. 2016 Mar 8;11(3):e0150460. doi: 10.1371/journal.pone.0150460 (PMC4783079; doi:10.1371/journal.pone.0150460)
Supplement: S1 File — (DOCX) [file pone.0150460.s001.docx]

Supporting Information

**Drug repositioning for cancer therapy using a chemical genomics approach based on large-scale drug-induced transcriptional signatures**

Haeseung Lee, Seungmin Kang, and Wankyu Kim*

Ewha Research Center for Systems Biology, Division of Molecular & Life Sciences, Ewha Womans University, Seoul, Korea

* To whom correspondence should be addressed. Tel: +82-2-3277-4132; Fax: +82-2-3277-6809; Email: wkim@ewha.ac.kr

Figure A. The compound set used in this work.

Figure B. The public HTS datasets are reasonably distinct.

Figure C. Performance evaluation using the public anti-cancer HTS dataset as a benchmark in total compound space.

Table A. List of PubChem HTS datasets for glioblastoma, lung cancer, and breast cancer.

Table B. The five clusters of similar pathway enrichment patterns with the active DR candidates and 15 other cancer drugs.


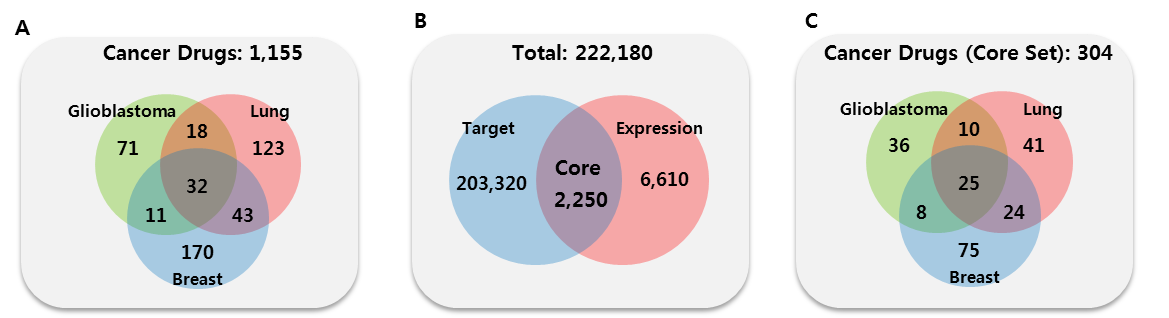


Figure A. The compound set used in this work. (A) The cancer drug set (CD set) and known drug set (KD set) for glioblastoma, lung cancer, and breast cancer. (B) The compound set with one or more known targets (T) and expression signature (E). Structural signatures (S) are available for all compounds. The core set consisted of 2,250 compounds with all three types of signatures (S, T, and E). (C) The CD set and KD set that are included in the core set.


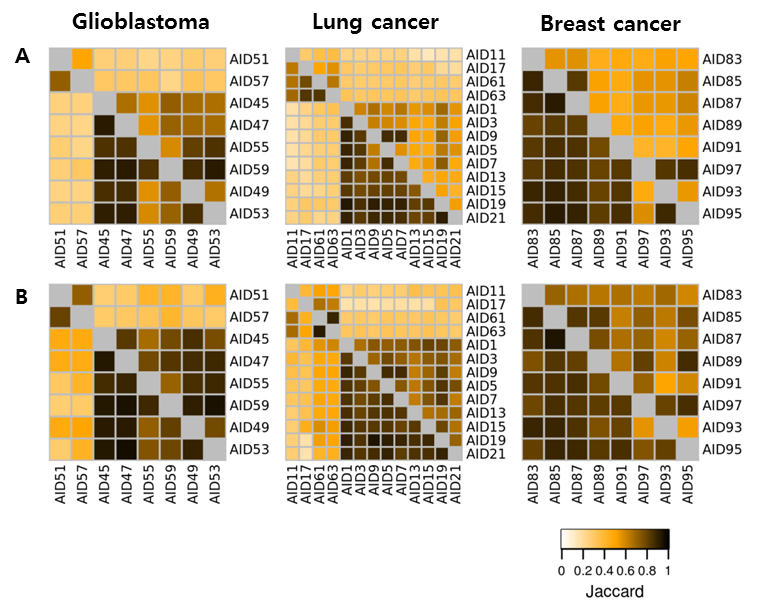


Figure B. The public HTS datasets are reasonably distinct. The redundancy among the public HTS datasets measured using the Jaccard index for (A) the hit compounds (upper diagonal) and total assayed compounds. (B) The same as (A) except that only the compounds in the core set were counted.


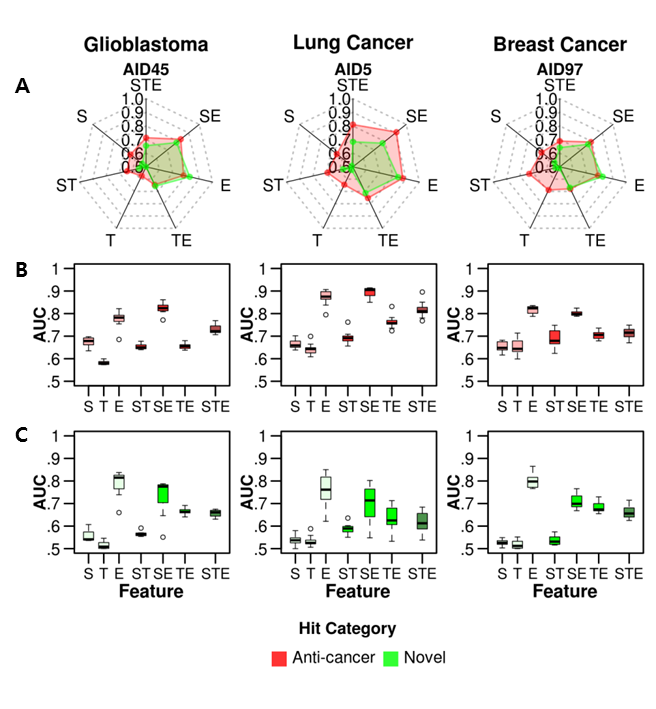


Figure C. Performance evaluation using the public anti-cancer HTS dataset as a benchmark in total compound space. The seven classifiers (S, T, E, ST, SE, TE, and STE) were evaluated based on the AUCs of the ROC curve for glioblastoma, lung cancer, and breast cancer. All assayed compounds were evaluated. The AUC values were calculated by averaging 100 rounds of 3-fold cross validation. (A) Typical examples of performance evaluation using the HTS data set for glioblastoma (AID45), lung (AID5), and breast cancer (AID97). The AUCs were independently calculated using two distinct sets of hit compounds as a benchmark (or positives)—*i.e.,* i) the hit compounds of known anti-cancer activity (red lines) and ii) novel hits (green lines). The distribution of AUCs using (B) the compounds of known anti-cancer activity as a benchmark, and (C) novel hits as a benchmark

Table A. List of PubChem HTS datasets for glioblastoma, lung cancer, and breast cancer.

| **AID** | **Hit compound** | **Test**  **compound** | **Hit**  **(Core)** | **Test**  **(Core)** | **Cell line** | **Cell type** |
| --- | --- | --- | --- | --- | --- | --- |
| AID45 | 2,054 | 40,818 | 33 | 219 | SF-268 | Glioblastoma |
| AID47 | 2,075 | 41,186 | 37 | 219 | SF-295 |  |
| AID49 | 2,119 | 38,553 | 37 | 214 | SF-539 |  |
| AID51 | 786 | 11,942 | 24 | 78 | XF 498 |  |
| AID53 | 1,730 | 40,717 | 33 | 220 | SNB-19 |  |
| AID55 | 2,101 | 38,474 | 40 | 215 | SNB-75 |  |
| AID57 | 591 | 13,466 | 18 | 78 | SNB-78 |  |
| AID59 | 2,219 | 41,196 | 41 | 220 | U251 |  |
| AID1 | 2,098 | 41,244 | 40 | 202 | NCI-H23 | Non-small cell lung cancer |
| AID3 | 1,857 | 37,995 | 32 | 193 | NCI_H226 |  |
| AID5 | 1,581 | 39,900 | 33 | 204 | NCI-H322M |  |
| AID7 | 2,422 | 39,900 | 40 | 201 | NCI-H460 |  |
| AID9 | 1,913 | 39,961 | 40 | 202 | HOP-62 |  |
| AID11 | 608 | 11,078 | 19 | 72 | HOP-18 |  |
| AID13 | 1,987 | 36,402 | 39 | 200 | HOP-92 |  |
| AID15 | 2,768 | 37,442 | 42 | 199 | NCI-H522 |  |
| AID17 | 730 | 13,394 | 21 | 53 | LXFL 529 |  |
| AID19 | 2,023 | 41,473 | 36 | 207 | A549/ATCC |  |
| AID21 | 1,482 | 40,250 | 31 | 202 | EKVX |  |
| AID61 | 909 | 13,208 | 26 | 78 | DMS 273 | Small cell lung cancer |
| AID63 | 948 | 14,286 | 26 | 81 | DMS 114 |  |
| AID83 | 2,361 | 28,597 | 40 | 189 | MCF7 | Breast cancer cell line |
| AID85 | 1,867 | 28,261 | 30 | 179 | MDA-MB-435 |  |
| AID87 | 1,820 | 27,662 | 30 | 178 | MDA-N |  |
| AID89 | 1,280 | 25,315 | 28 | 159 | BT0549 |  |
| AID91 | 1,556 | 26,679 | 31 | 173 | T-47D |  |
| AID93 | 1,472 | 28,546 | 30 | 187 | NCI/ADR-RES |  |
| AID95 | 1,454 | 27,770 | 27 | 175 | MDA_MB_231 |  |
| AID97 | 1,464 | 26,619 | 31 | 170 | HS 578T |  |

Table B. The five clusters of similar pathway enrichment patterns with the active DR candidates and 15 other cancer drugs.

| **DR candidates**  **(cluster)** | **Enriched Pathways** | | **Cancer Drugs**  **In the same cluster** | **Target cancer type** | **Mode of Action** |
| --- | --- | --- | --- | --- | --- |
| Maprotiline,  Nortriptyline  (I) | Up | Steroid synthesis  Immune system  Ribosome | Crizotinib | non-small cell lung carcinoma | ALK and ROS1 inhibitor |
|  |  |  | Fulvestrant | breast | estrogen receptor inhibitor |
|  |  |  | Vorinostat | lymphoma | histone deacetylases inhibitor |
|  | Down | Cell cycle, DNA repair Energy metabolism P53 signaling pathway  Ribosome | Tamoxifen | breast | estrogen receptor inhibitor |
|  |  |  | Saracatinib | broad cancers | c-Src/Abl kinase inhibitor |
|  |  |  | Toremifene | breast | estrogen receptor inhibitor |
|  |  |  | Sunitinib | kidney and gastrointestinal tract | receptor tyrosine kinase inhibitor |
| Astemizole  Amlodipine  (II) | Up | Steroid synthesis | Cediranib | Liver, lung, colon cancer  (+ other solid tumors) | VEGFR inhibitor |
|  | Down | Cell cycle, DNA repair |  |  |  |
| Mometasone  (III) | Up | -- | Trametinib | melanoma | MEK inhibitor |
|  | Down | Cell cycle, DNA repair | Exemestane | breast | aromatase inhibitors |
| Ivermectin  (IV) | Up | Aminoacid metabolism Aminoacyl-tRNA syntesis | Dabrafenib | melanoma | BRAF and CRAF inhibitor |
|  |  |  | Azacitidine | broad cancers | DNA methyltransferase inhibitor |
|  | Down | Cell cycle, DNA repair Steroid synthesis P53 signaling pathway | Sorafenib | kidney | Raf kinase inhibitor |
|  |  |  | Vemurafenib | melanoma | BRAF inhibitor |
| Trifluridine  (V) | Up | P53 pathway | Axitinib | kidney, breast and pancreas | VEGFR inhibitor |
|  | Down | Cell cycle |  |  |  |
